# Comparative phosphoproteomic analysis of blast resistant and susceptible rice cultivars in response to salicylic acid

**DOI:** 10.1186/s12870-019-2075-5

**Published:** 2019-10-28

**Authors:** Ranran Sun, Shiwen Qin, Tong Zhang, Zhenzhong Wang, Huaping Li, Yunfeng Li, Yanfang Nie

**Affiliations:** 1 Guangdong Province Key Laboratory of Microbial Signals and Disease Control, South China Agricultural University, Guangzhou, 510642 China; 2 College of Agriculture, South China Agricultural University, Guangzhou, 510642 China; 3 Research Center of Perennial Rice Engineering and Technology in Yunnan, Yunnan University, Kunming, 650500 China; 4 College of Materials and Energy, South China Agricultural University, Guangzhou, 510642 China

**Keywords:** Salicylic acid, Rice, Phosphoproteome, Two-dimensional gel electrophoresis, Protein phosphorylation

## Abstract

**Background:**

Salicylic acid (SA) is a significant signaling molecule that induces rice resistance against pathogen invasion. Protein phosphorylation carries out an important regulatory function in plant defense responses, while the global phosphoproteome changes in rice response to SA-mediated defense response has not been reported. In this study, a comparative phosphoproteomic profiling was conducted by two-dimensional gel electrophoresis (2-DE) and mass spectrometry (MS) analysis, with two near-isogenic rice cultivars after SA treatment.

**Results:**

Thirty-seven phosphoprotein spots were differentially expressed after SA treatment, twenty-nine of which were identified by MALDI-TOF/TOF MS, belonging to nine functional categories. Phosphoproteins involved in photosynthesis, antioxidative enzymes, molecular chaperones were similarly expressed in the two cultivars, suggesting SA might alleviate decreases in plant photosynthesis, regulate the antioxidant defense activities, thus improving basal resistance response in both cultivars. Meanwhile, phosphoproteins related to defense, carbohydrate metabolism, protein synthesis and degradation were differentially expressed, suggesting phosphorylation regulation mediated by SA may coordinate complex cellular activities in the two cultivars. Furthermore, the phosphorylation sites of four identified phosphoproteins were verified by NanoLC-MS/MS, and phosphorylated regulation of three enzymes (cinnamoyl-CoA reductase, phosphoglycerate mutase and ascorbate peroxidase) was validated by activity determination.

**Conclusions:**

Our study suggested that phosphorylation regulation mediated by SA may contribute to the different resistance response of the two cultivars. To our knowledge, this is the first report to measure rice phosphoproteomic changes in response to SA, which provides new insights into molecular mechanisms of SA-induced rice defense.

## Background

Rice (*Oryza sativa* L.) is an economically important cereal crop throughout the world, providing food for over 50% of global population [1]. The ascomycetous fungus *Magnaporthe oryzae* causes the rice blast, one of the most devastating fungal diseases in rice production and thus poses a great threat to the world’s food security [2]. Thus far, the disease control is mainly based on using fungicides and breeding resistant cultivars. However, fungicide could fail to satisfy the requirement of environment and human health regulations, and resistant cultivars could be overcome by the quick arising/evolving of new races of *M. oryzae* [3, 4]. As an important signaling molecule, salicylic acid (SA) can induce plant resistance against multiple fungal, viral and bacterial pathogens [5]. SA-mediated plant defense responses are actively involved in both PTI (PAMP-triggered immunity) and ETI (effector-triggered immunity) [6]. In the previous study, we confirmed that SA can protect rice against infection by *M. oryzae* race ZC_13_ in CO39 (susceptible cultivar) and in a near isogenic line C101LAC, which carries the resistance gene *Pi-1* against *M. oryzae* race ZC_13_ and thus represents a resistant cultivar [7]. Proteomic analysis further showed that SA coordinates multiple cellular activities to facilitate defense response and recovery in both rice cultivars. However, it awaits further elucidation about the detailed molecular mechanisms of SA-induced rice defense response against *M. oryzae*.

Phosphorylation is one of the most important post-translational protein modifications (PTMs), regulating a wide range of cellular functions in various organisms, including cell signaling, metabolism, stress responses and defense responses [8]. Phosphoproteomics can capture the dynamics and specificity of protein phosphorylation, and therefore enhance our understanding of fundamentals and complex biological processes [9]. In recent years, a large number of emerging evidences suggested that protein phosphorylation can regulate plant stress responses triggered by exogenous hormone and biotic stress [10–13]. It has been also shown that protein phosphorylation was involved in the activation of SA-induced plant resistance using traditional biochemical methodologies [14, 15]. To our knowledge, the general scope of such connections between protein phosphorylation events and SA-induced rice resistance has not been studied.

In the present study, we performed a comparative phosphoproteome to reveal the detail SA-induced mechanism in rice using two-dimensional gel electrophoresis (2DE), Pro-Q diamond phosphoprotein stain and MALDI-TOF/TOF mass spectrometer. Thirty-seven SA-responsive phosphoprotein spots were found and twenty-nine of them were identified. Phosphoproteins involved in similar or different function and expression patterns in resistant and susceptible rice cultivars were discussed. The results provided new insights about the dynamic phosphoproteomes at different time points in rice upon SA treatment, and broadened the understanding of SA-mediated rice resistance against *M. oryzae* infection via regulation on protein phosphorylation.

## Results

### Specificity analysis of MOAC-enriched putative phosphoproteins from rice leaves

**Fig. 1:**
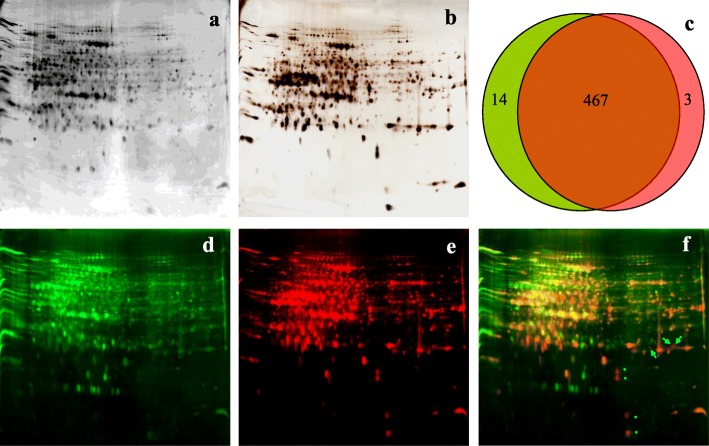
2DE analysis of MOAC-enriched putative phosphoproteins from rice leaves. Original-color image of the same 2DE gel was stained by **a** Pro-Q diamond and **b** silver nitrate. **c** Venn diagram analysis of MOAC-enriched putative phosphoproteins in 2DE gels that overlapped between Pro-Q Diamond staining (green) and silver staining (red). False-color images of 2DE gels were visualized with different colors using PDQuest software; **d** Pro-Q diamond-stained protein spots were colored green and **e** silver-stained protein spots were colored red. **f** An overlay of the two images (**d** and **e**). The phosphoprotein spots were appeared yellow, and the non-phosphoprotein spots were appeared red (as shown by arrows). Asterisks indicate spots that were very abundant in the silver image but lightly stained in the Pro-Q Diamond image

A total of 490 ± 15 μg putative phosphoproteins were enriched from 8 mg of total proteins. To test the specificity of MOAC for phosphoproteins, MOAC-enriched putative phosphoproteins were separated by 2DE and sequentially stained for phosphoproteins using Pro-Q Diamond, and for total proteins by silver stain (Fig. 1a, b). 481 ± 9 protein spots could be detected on Pro-Q Diamond-stained gels and 469 ± 12 spots on sequential silver-stained gels; of these spots, 466 were common to the two staining methods (Fig. 1c). To determine the specificity of MOAC-enriched phosphoproteins, the sequential staining images were overlapped and the protein spots were visualized in different colors using PDQuest software (Fig. 1d, e, f). The overlay image showed that most of the protein spots (over 99%) appeared in yellow, indicating that these proteins were phosphoproteins; only three protein spots appeared in red, indicating that these were nonphosphoproteins (Fig. 1f). The results demonstrated the MOAC is selective enough for detecting rice leaf phosphoproteins.

### Phosphoproteome changes in rice leaves upon SA treatment

To study the changes of SA-induced phosphoprotein profiles, we conducted a 2DE-based phosphoproteomic analysis at 12 h and 24 h after SA treatment of C101LAC and CO39. In our previous work, we conducted SA-induced rice resistance against *M. oryzae* at different concentrations ranging from 0.01 mM to 1 mM SA [7]. The results showed that the optimum concentration of SA treatment to induce blast resistance of rice seedlings was at 0.1 mM. Thus, 0.1 mM SA was used in this study. At least three independent 2DE analysis was performed for each treatment, with a high level of reproducibility. Eight representative gels and other replicate gels were shown in Additional file 1: Figure S1 and Figure S2, respectively (Additional file 1). Fold changes above 1.5 in all three replicates were used as thresholds to determine the SA-responsive phosphoproteins. Additionally, differential expression pattern was shown to be similar in all three replicates, and we manually checked all the spots to ensure confidence in differentially regulated phosphoproteins. A total of 37 SA-responsive phosphoproteins were obtained from the two cultivars (Additional file 1: Figure S1). For each cultivar, 30 and 28 SA-responsive phosphoprotein spots were detected in CO39 and C101LAC, respectively; 21 of which were common in these two cultivars. A close-up view of the SA-responsive phosphoproteins on the 2DE gels was shown in (Additional file 1: Figure S3). The relative intensities of SA-responsive phosphoproteins were displayed in (Additional file 1: Fig. S4). We conclude that SA treatment resulted in reproducible and significant changes to these protein spots, which we could investigate further.

### MALDI-TOF/TOF identification of SA-responsive phosphoproteins

The 37 SA-responsive phosphoprotein spots were excised from 2DE gels and further identified by MALDI-TOF/TOF MS. Of these, 29 phosphoproteins were identified with high confidence (Table 1), while the remaining 8 phosphoprotein spots (2, 10, 19, 22, 23, 26, 32 and 35) did not show a good match in the database. The spectra of protein spot 11 were provided as an example of analysis (Additional file 1: Figure S5). Generally, one protein spot in 2DE gel represented one unique protein. However, we noted that three phosphoproteins were identified in more than one spot in the same gel (Table 1, Additional file 1: Figure S1). For example, three phosphoprotein spots (11, 12 and 13) were identified as probable glutamyl endopeptidase, two (17 and 18) as glyceraldehyde-3-phosphate dehydrogenase, two (24 and 25) as alpha 1,4-glucan phosphorylase. Consistent with our results, it has been reported that protein isoforms migrated as a chain of spots, most likely due to post-translational modifications [16]. Proteins with multiple phosphorylation states could also lead to electrophoresis patterns that multiple spots are with similar molecular weight but different p*I* [17].

**Table 1:** SA-responsive rice phosphoproteins identified by MALDI-TOF/TOF MS

Protein Spot No.^a^	Protein Name	Uniprot Accession No.	Molecular Mass (kDa)	Isoelectric Point	Pep. Count^b^	Protein Score^c^	Protein Score (C.I.%)^d^	Relative fold changes^e^	
								CO39	C101LAC
Photosynthesis									
1	HAD-superfamily hydrolase, subfamily IA, variant 3 containing protein, expressed	Q10I42	34.1	8.4	11	560	100	NC	↓ 12 h 1.93 ± 0.01
5	Rubisco activase, chloroplast precursor, putative, expressed	H2KWJ8	39	5.4	19	761	100	↑ 12 h 4.06 ± 0.32	↑ 12 h 4.43 ± 0.53
20	Rubisco large subunit	POC511	26	7.0	12	452	100	↑ 12 h 1.83 ± 0.17	↑12 h 1.94 ± 0.14
27	Putative transketolase 1	Q5VNW1	69.4	5.4	22	521	100	↑ 24 h 1.53 ± 0.02	↑ 24 h 1.90 ± 0.02
34	Chloroplast 23 kDa polypeptide of photosystem II	B0FFP0	20	5.6	11	755	100	↓ 24 h 2.89 ± 0.01	NC
Defense-related protein									
37	Putative cinnamoyl-CoA reductase	Q69U05	36	5.7	16	398	100	NC	↑24 h 1.63 ± 0.026
Antioxidative enzymes									
15	GDP-mannose 3,5-epimerase 1	A3C4S4	43	5.8	23	702	100	↓12 h 2.36 ± 0.02	NC
33	L-ascorbate peroxidase 1	B7E6Z4	27.3	5.4	14	496	100	↓24 h 1.93 ± 0.01	↓24 h 1.83 ± 0.03
Molecular chaperone									
23	Putative chaperonin 60 beta	Q9LWT6	64	5.6	23	560	100	↑ 24 h 3.71 ± 0.17	↑ 24 h 2.20 ± 0.01
Protein synthesis and degradation									
4	ATP-dependent Clp protease proteolytic subunit	Q6H7I9	32	6.7	5	328	100	↑ 12 h 1.59 ± 0.04	↑12 h 1.63 ± 0.03
6	Alpha/beta hydrolase	Q710Q1	18	8.9	8	415	100	↓ 12 h 2.24 ± 0.07	↑ 12 h 3.01 ± 047
11	Probable glutamyl endopeptidase, chloroplastic	Q10MJ1	104.48	5.7	26	621	100	NC	↑ 12 h 1.75 ± 0.02
12	Probable glutamyl endopeptidase, chloroplastic	Q10MJ1	104.48	5.7	26	621	100	↑ 12 h 1.70 ± 0.01	↑ 12 h 2.08 ± 0.28
13	Probable glutamyl endopeptidase, chloroplastic	Q10MJ1	104.48	5.7	26	621	100	NC	↑ 12 h 2.08 ± 0.08
28	Eukaryotic initiation factor 4A-1	P35683	47.3	5.4	23	719	100	↓ 24 h 1.61 ± 0.02	↑ 24 h 1.78 ± 0.03
30	Elongation factor Tu	Q6ZI53	51	6.2	21	608	100	↓ 24 h 1.62 ± 0.09	NC
Carbohydrate metabolism									
9	Phosphoglycerate mutase	Q5QMK7	61	5.4	18	386	100	NC	↓ 12 h 2.53 ± 0.38
17	Glyceraldehyde-3-phosphate dehydrogenase	A2XC18	47.5	6.2	16	652	100	↑12 h 2.67 ± 0.17	↓ 12 h 2.99 ± 0.02
18	Glyceraldehyde-3-phosphate dehydrogenase	A2XC18	47.5	6.2	16	652	100	↑12 h 2.19 ± 0.05	↓ 12 h 2.89 ± 0.02
24	Alpha 1,4-glucan phosphorylase	Q9ATK9	105	5.4	24	508	100	↑24 h 1.58 ± 0.04	NC
25	Alpha 1,4-glucan phosphorylase	Q9ATK9	105	5.4	24	508	100	↑24 h 2.47 ± 0.29	↑ 24 h 1.87 ± 0.03
31	Phosphoribulokinase	Q6Z8F4	44.9	5.7	15	830	100	↓ 24 h 15.98 ± 0.55	↑ 24 h 3.60 ± 0.42
32	Phosphoribulokinase	Q8GRU9	44.9	5.7	18	624	100	↓ 24 h 2.07 ± 0.01	↑ 24 h 2.25 ± 0.03
Amino acid metabolism									
16	Aspartate aminotransferase	Q84V24	46	5.9	25	859	100	↓12 h 2.09 ± 0.03	NC
36	Cysteine synthase	Q2QLX5	43.8	8.8	18	479	100	NC	↑24 h 2.04 ± 0.05
Energy metabolism									
21	ATP synthase epsilon chain, chloroplastic	P0C2Z3	15.3	5.0	9	559	100	NC	↓12 h 1.82 ± 0.02
Metabolism									
7	Probable bifunctional riboflavin biosynthesis protein RIBA 1, chloroplastic	Q6Z234	59.2	5.6	18	306	100	↑ 12 h 1.62 ± 0.13 ↑ 24 h 2.74 ± 0.10	↑ 24 h 2.87 ± 0.08
8	Probable bifunctional riboflavin biosynthesis protein RIBA 1, chloroplastic	Q6Z234	59.2	5.6	18	306	100	↑12 h 2.21 ± 0.09	↓12 h 3.15 ± 0.15
29	Glucose-1-phosphate adenylyltransferase large subunit 3, chloroplast, putative, expressed	Q6AVT2	55.8	7.0	31	844	100	↓ 24 h 1.68 ± 0.09	↑ 24 h 3.46 ± 0.09

^a^Spot numbers are according to 2DE gels as shown in Fig. 1

^b^Number of unique peptides matched to mass peaks

^c^MS and MS/MS combined score

^d^C.I. % for the protein score rates the confidence level of the Protein Score

^e^The fold changes listed in the table were derived by comparison of relative protein intensity between SA-treated and control, which were calculated with PDQuest 8.0 software. The detailed information of relative protein intensity was listed in Additional file 1: Fig. S2. Only the proteins with changes ≥1.5-fold were listed. “NC” indicates the proteins with no significant changes (<1.5-fold). Differentially regulated phosphoproteins were marked by arrow, which indicates up-(↑) or down-regulated (↓) phosphoproteins in response to SA treatment. The number with no parentheses indicated the fold changes of differentially regulated phosphoproteins in 2DE gels

Based on biological annotations from UniProtKB database (www.uniprot.org), the 29 identified SA-responsive phosphoproteins were functionally classified into 9 groups: photosynthesis, defense, antioxidative enzymes, protein synthesis and degradation, molecular chaperones, amino acid metabolism, carbohydrate metabolism, energy metabolism and other metabolism (Additional file 1: Figure S6). Among them, phosphoproteins involved in carbohydrate metabolism, protein synthesis and degradation were the most abundant, both representing 24.14% (7/29) of the phosphoproteins identified, respectively.

### Phosphorylation site identification

**Fig. 2:**
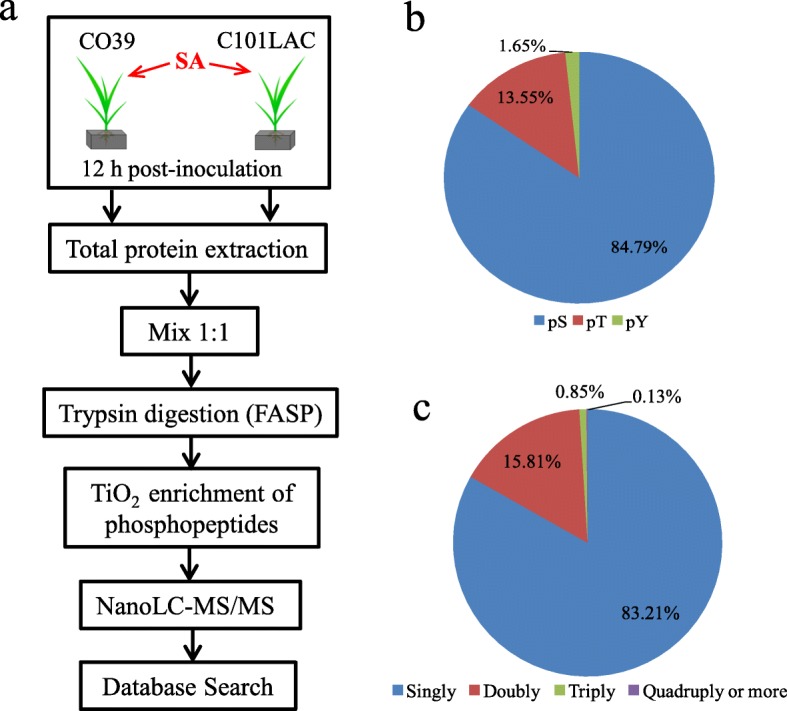
NanoLC-MS/MS identification for phosphorylation sites of total proteins from rice leaves. **a** Overview of the experimental design for the phosphorylation patterns of total proteins. The total proteins were digested with trypsin. Phosphopeptides are enriched from the pooled peptide mixture with titanium dioxide (TiO_2_) beads and subsequently analyzed with nanoLC−MS/MS. **b** The distribution of peptides having one, two, three, and four and more phosphorylation sites. **c** The distribution of phosphorylated residues. pS, phosphoserine; pT, phosphothreonine; pY, phosphotyrosine

Next, we tried to determine the phosphorylation patterns of total proteins by using NanoLC-MS/MS analysis, following the workflow as depicted in Fig. 2a. Totally, 1815 phosphosites were identified, which come from 1537 phosphopeptides (Additional file 2: Table S1) on 839 phosphoproteins (Additional file 3: Table S2). Among these 1815 phosphorylated residues, there were 1539 phosphoserine (pS), 246 phosphothreonine (pT), and 30 phosphotyrosine (pY), corresponding to 84.79, 13.55, and 1.65% respectively of all phosphorylated residues (Fig. 2b). We noticed that 257 phosphopeptides were multiply phosphorylated, and 1279 phosphopeptides were singly phosphorylated (Fig. 2c). Then, the 29 identified SA-responsive phosphoproteins were scanned in the NanoLC-MS/MS analysis data. Phosphorylation sites within 4 identified SA-responsive phosphoproteins were verified (Additional file 4: Table S3). The MS/MS spectrum of a representative phosphorylated peptide (ELLS*YEYDGDEVPIVAGSALK), Elongation factor Tu (spot 30) was shown in (Additional file 1: Figure S7), as an example.

### Enzyme activities in rice leaves induced by SA treatment

**Fig. 3:**
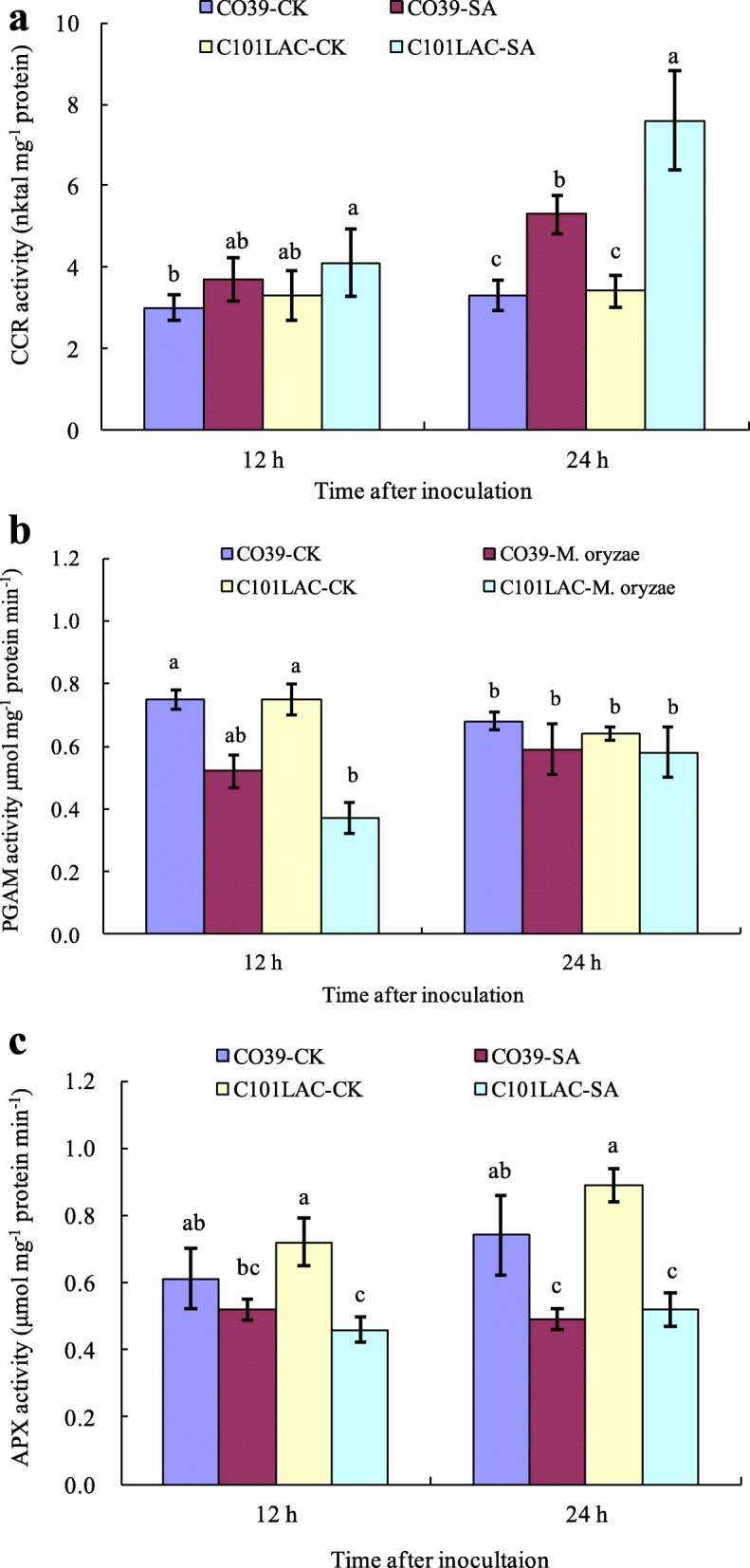
Quantitative analysis of **a** CCR, **b** PGAM, and **c** APX activities in the leaves of rice seedlings after SA treatment. Bars indicate ± standard error of the mean. Different small letters in each group indicate significant differences at *P* ≤ 0.05

To further validate possible regulation of enzyme activity by protein phosphorylation, we selected three important enzymes from the identified phosphoproteins, and assessed their activities. A significant increase in cinnamoyl-CoA reductase (CCR) activity was detected in C101LAC at 24 h after SA treatment (Fig. 3a). CCR showed an increased phosphorylation level in C101LAC at 24 h after SA treatment, suggesting that the enzyme activity of CCR was regulated by phosphorylation. Consistent with the phosphoproteomic results, a significant decrease in APX activity was noted in both cultivars 24 h after SA treatment. A significant decrease in PGAM activity was detected only in C101LAC 12 h after SA treatment, but little change was found in CO39 (Fig. 3b, c). Phosphoproteomic analysis also showed that phosphorylated PGAM and phosphorylated APX were down-regulated only in C101LAC at 12 h after SA treatment, but little change was found in CO39 (Table 1). These results above strongly suggesting that the enzyme activity of PGAM and APX was regulated by phosphorylation.

### ROS accumulation in rice leaves induced by SA treatment

**Fig. 4:**
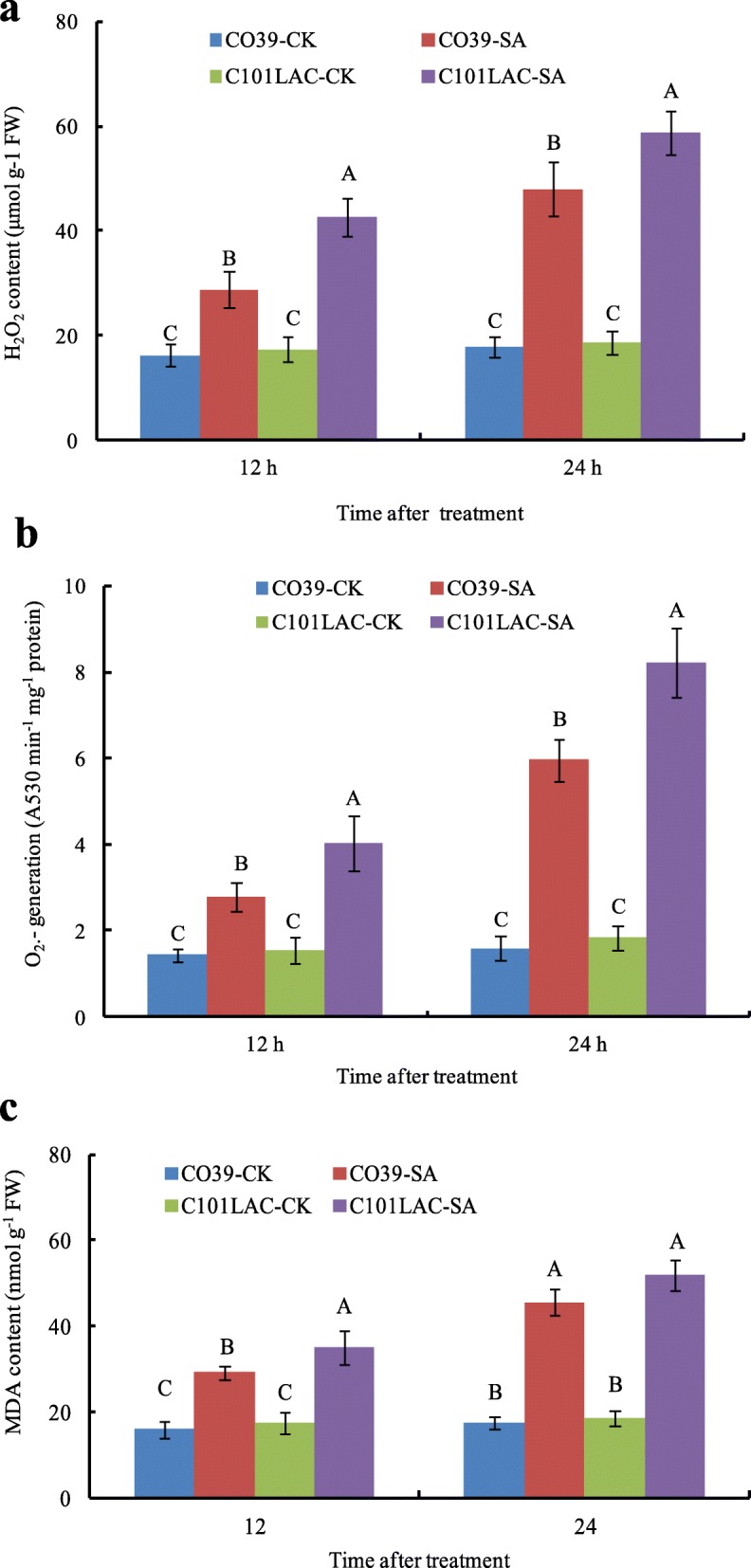
ROS production in rice leaves induced by SA. Water was used as a control (CK). **a** O_2_^.-^ production. **b** H_2_O_2_ content. **c** MDA contents. Bars indicate ± standard error of the mean. Different capital letters in each group indicate significant differences at *P* ≤ 0.01

SA and ROS production in rice defense response to biotic and abiotic stress has been well documented [18, 19]. In this study, the phosphoproteomic analysis revealed several phosphoproteins involved in biogenesis of ROS. In agreement with the phosphoproteomic results, a significant increase in O_2_^.-^, H_2_O_2_ and MDA contents was noted in both rice cultivars after SA treatment, compared with the corresponding controls (Fig. 4). Relative to the control, SA resulted in an increase of H_2_O_2_ contents by 1.77 and 2.72 times in CO39, 2.47 and 3.15 times in CO101LAC at 12 h and 24 h, respectively (Fig. 4a). An increase of O_2_^.-^ content was also noted by 1.94 and 3.75 times in CO39, 2.59 and 4.46 times in CO101LAC at 12 h and 24 h after SA treatment, respectively (Fig. 4b). MDA contents was significantly increased by 1.83 and 2.61 times in CO39, 2.61 and 2.79 times in CO101LAC 12 h and 24 h post SA treatment, respectively, compared with the controls (Fig. 4c). Taken together, SA treatment significantly increased the contents of O_2_^.-^, H_2_O_2_ and MDA in both resistant (C101LAC) and susceptible (CO39) cultivars, but the fold changes in C101LAC were significantly higher than that in CO39.

### Transcriptional expression analysis of SA-responsive phosphoproteins

**Fig. 5:**
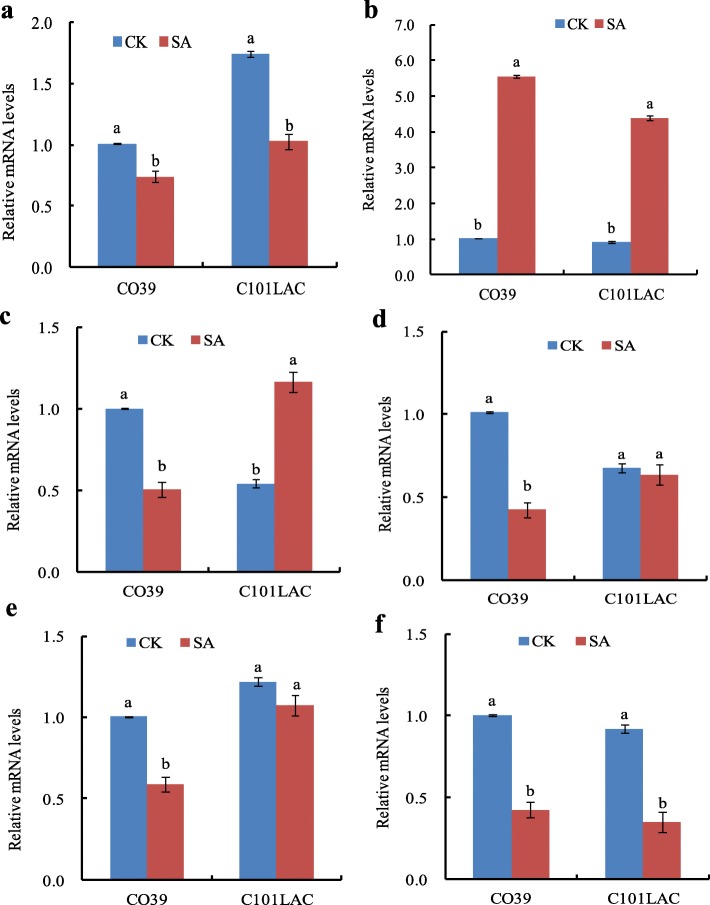
Transcript analysis by qRT-PCR of six differentially expressed genes after SA treatment. **a** Putative chaperonin 60 beta, **b** Elongation factor Tu, **c** Eukaryotic initiation factor 4A-1, **d** Phosphoribulokinase, **e** Glyceraldehyde-3-phosphate dehydrogenase, and **f** L-ascorbate peroxidase 1. Bars indicate ± standard error of the mean. Different small letters in each group indicate significant differences at *P* ≤ 0.05

Six genes encoding the identified phosphoproteins (Additional file 4: Table S4) were selected for expression analysis via qRT-PCR. The gene encoding putative chaperonin 60 beta was significantly down-regulated in CO39 after SA treatment, but up-regulated in C101LAC (Fig. 5a). Elongation factor Tu significantly decreased in CO39 at mRNA levels, did not significantly vary in C101LAC (Fig. 5b). The expression changes of the above two phosphoproteins at mRNA levels were consistent with the 2DE results both in CO39 and C101LAC. Two phosphoproteins (eukaryotic initiation factor 4A-1 and phosphoribulokinase) showed a significant decrease in mRNA levels but a significant increase in phosphorylation levels in C101LAC; however, they decreased both in mRNA levels and phosphorylation levels in CO39 (Fig. 5c, d). Interestingly, glyceraldehyde-3-phosphate dehydrogenase significantly decreased in mRNA levels, but increased in phosphorylation levels in CO39; however, the expression decreased both in mRNA levels and phosphorylation levels in C101LAC (Fig. 5e). L-ascorbate peroxidase 1 significantly increased in mRNA levels both in CO39 and C101LAC; while its phosphorylation level showed a typical decrease in C101LAC and no significant variation in CO39 (Fig. 5f). In total, these data showed a lack of correlation between transcriptional regulation and post-tranlational regulation (by protein phosphorylation) in rice plants treated with SA. Consistent with this results, several reported phosphoproteomics studies had also revealed different changes in the mRNA levels and their corresponding proteins levels [20, 21]. The results highlighted the importance of SA-induced resistance mechanism at multiple molecular levels in rice plant.

## Discussion

SA is an important signaling molecule that plays key roles in the regulation of plant defense against pathogens. In our previous work, we explored the molecular mechanisms of SA-mediated protection of rice against *M. oryzae* infection by proteomic profiling analysis with blast-resistant vs. -susceptible rice cultivars after SA treatment [7]. Protein phosphorylation, as one of the most common and best characterized post-translational modifications, is a key process that regulates a large number of biological processed in plants, including cell signaling, metabolism, hormone and stress responses [22]. However, it is very difficult to detect phosphoproteins by the “proteomic” approach, due to the low abundance of phosphoproteins [23]. For further understanding the molecular mechanisms, we performed a comparative phosphoproteome analysis of two near rice isogenic lines after SA treatment. In total, we identified 29 phosphoproteins in response to SA treatment, belonging to 9 functional categories. Among the 29 phosphoproteins, 17 protein spots were common in both resistant (C101LAC) and susceptible (CO39) cultivars, suggesting that some physiological processes could be commonly influenced by SA. However, the other 12 phosphoproteins showed different changes between CO39 and C101LAC. The expression patterns of these phosphoproteins could help to understand the different molecular mechanism of SA-induced resistance against *M. oryzae* in the two rice cultivars.

Photosynthesis has been well-known to be highly sensitive to various exogenous stimulus, including SA, JA and some plant hormone [24, 25]. In this study, total 5 phosphoproteins related to photosynthesis were differentially regulated after SA treatment, in which chloroplast 23 kDa polypeptide of photosystem II (PSII, spots 34) was firstly reported to be phosphorylated which was validated by NanoLC-MS/MS. It has been previously demonstrated that photosynthesis could be regulated through phosphorylation of photosynthesis-related proteins. For example, phosphorylation of rubisco activase (RCA), known as an ancillary photosynthetic protein essential for Rubisco activity, was reduced following abiotic stress, resulting in the malfunctioning of photosynthesis [26]. Transketolase (TKL), a key enzyme linking the non-oxidative pentose phosphate pathway and the Calvin cycle, is presumed to participate in the functional regulation of numerous metabolic pathways by phosphorylation [27]. Previous studies have shown that dephosphorylation leads to a dramatic decrease in the TKL activity, which declines the rates of photosynthesis [28]. In this study, most of the phosphoproteins, including RCA (spots 5), Rubisco large subunit (Spots 20) and TKL (spots 27) were up-regulated in both rice cultivars at 12 h and 24 h post SA treatment. Consistent with our results, it has previously been reported that SA could alleviate decreases in plant photosynthesis against pathogen infection to produce various metabolites and energy, thus improving plant defense response [7]. However, we also noted that HAD-superfamily hydrolase (subfamily IA, variant 3 containing protein, spot 1) were down-regulated in C101LAC 12 h post SA treatment. It is suggested that SA regulates plant photosynthesis via phosphorylation of photosynthesis-related proteins and the effect of SA on the photosynthetic machinery is complex in rice plants.

We identified a putative cinnamoyl-CoA reductase (CCR, spot 37) as SA-responsive phosphoprotein. CCR catalyzes the first step of the monolignol pathway for lignin biosynthesis and therefore plays an essential role in defense-related processes in rice [29]. In our previous study, CCR was firstly reported to be dephosphorylated in susceptible rice plants after *M. oryzae* infection, which resulted in a decrease in enzyme activities [30]. In this study, CCR was notably up-regulated only in C101LAC at 24 h after SA treatment (Table 1). Correspondingly, CCR activity was significantly increased in the resistant cultivar at 24 h after SA treatment, but not in the susceptible cultivar (Fig. 3a). CCR, as an effector of small GTPase Rac in rice defense signaling, is considered to be activated by OsRac1, which further leads to efficient production of monolignols, deposition of lignin, increased ROS accumulation [31]. Taken together, we speculate that posphorylation of CCR may increase enzyme activities, accelerate lignin synthesis and ROS accumulation, thus further activate rice defense responses against infection of pathogens.

Phosphorylation of antioxidant enzymes has been reported to be involved in antioxidant defense [32]. In this study, two phosphoproteins were identified as GDP-mannose 3,5-epimerase 1 (OsGME 1, spot 15) and L-ascorbate peroxidase 1 (OsAPx01, spot 33), which are known as antioxidant enzymes. Phosphorylated OsGME 1 was down-regulated only in the susceptible cultivar at 12 h after SA treatment, and phosphorylated OsAPx01 was down-regulated in both cultivars. Previous studies have showed that dephosphorylation decreases the activities for APX and OsGME 1 upon biotic or abiotic stress in several plants [26, 33]. To validate phosphorylated regulation of enzyme activity, APX activity was performed (Fig. 3b). The results showed that APX activity was significantly decreased in both cultivars at 24 h after SA treatment, which were consistent with the phosphoproteomic analysis. It has been suggested that down-regulation of scavenging/antioxidant systems and/or activities may contribute to increase ROS accumulation [34]. Earlier study reported that plant cells presumably regulated ROS levels by coordinating activities of ROS-generating enzymes such as SOD and ROS-degrading enzymes such as APX, POX, and CAT [35]. A detailed comparison of the influence of SA on H_2_O_2_ production, SOD activities and H_2_O_2_-degrading enzymes showed that tobacco leaves treated with SA may have enhanced H_2_O_2_ largely by activating enzymes capable of generating H_2_O_2_, and by inactivating enzymes that are capable of degrading H_2_O_2_ [36]. In this study, the findings that phosphorylation of APX and OsGME 1 was down-regulated in rice suggests a significant decrease of the activities of ROS-scavenging antioxidant enzymes, further accelerates oxidative burst in both cultivars. Consistent with this notion, significant ROS accumulation was observed in both rice cultivars after SA treatment (Fig. 4), which indicated that SA could regulate antioxidant enzymes’ activity by phosphorylation, then enhance ROS accumulation in rice plants as a defense response. However, SA and ROS interactions are complicated and awaits further investigation in future to fully elucidate.

One phosphoprotein was identified as 60 kDa chaperonin beta (Cpn60β, spot 23), which is a molecular chaperone involved in protein destination and assembly. Molecular chaperones play regulatory roles in prevention of stress injury, immune response, and cell death in plants, which might help plant enhance defense system against pathogen infection [37]. Phosphorylation is a common regulatory mode for the function of different classes of molecular chaperones, which markedly improves the capacity to enhance its binding to unfolded proteins, facilitate rapid degradation of certain abnormal proteins, and protect against oxidative stress injury [38, 39]. In this study, phosphorylated Cpn60β was up-regulated at 24 h after SA treatment in both cultivars (Table 1). A previously proteomic study showed that 60 kDa chaperonin was significantly up-regulated in resistant tomato cultivar compared with susceptible tomato cultivar after bacterial infection, which might provide enhanced defense system against *Pseudomonas solanacearum* [40]. Taken together, we speculate that SA-induced phosphorylation of Cpn60β may improve the activities of Cpn60β, further contributing towards rice defense against pathogen attack.

Seven phosphoproteins were involved in carbohydrate metabolism, including phosphoglycerate mutase (PGAM, spot 9), glyceraldehyde-3-phosphate dehydrogenase (GAPDH, spot 17 and 18), alpha 1,4-glucan phosphorylase L isozyme (α-GP, spots 24 and 25), phosphoribulokinase (PRK, spot 31 and 32). PGAM catalyzes the conversion of 3-phosphoglycerate to 2-phosphoglycerate, which is a crucial step in glycolysis. Previous phosphoproteomics studies showed that PGAM can be phosphorylated at multiple tyrosine sites, which enhances PGAM activity and upregulates glycolysis [41, 42]. In this study, phosphorylated PGAM was down-regulated only in C101LAC 12 h after SA treatment. To validate regulation of enzyme activity by SA-induced protein phosphorylation, PGAM activity was assessed (Fig. 3b). Agreeable with the phosphoproteomic analysis, the results showed that a significant decrease in PGAM activity was noted in C101LAC 12 h after SA treatment, but little change was found in CO39. α-GP catalyzes the reversible phosphorolysis of glucan chains and releases glucose-1-phosphate for starch resynthesis. Phosphorylated α-GP is active, and dephosphorylated α-GP is inactive [43]. GAPDH catalyzes a critical step in glycolysis; GAPDH activity was significantly decreased if phosphorylated [44]. In this study, phosphorylated α-GP was up-regulated both in CO39 and C101LAC 12 h after SA treatment, suggesting that SA might increase α-GP activity. However, phosphorylated GAPDH was down-regulated in C101LAC, but up-regulated in CO39 12 h after SA treatment. The results above suggest that accumulation of carbohydrates was differently regulated in two different rice cultivars by SA induction, indicating that carbohydrate metabolism pathways may be regulated by reverse phosphorylation of important enzymes. However, little is known about the potential role for phosphorylated regulation in these enzymes of carbohydrate metabolism and further investigation is needed to decipher their role in rice.

## Conclusions

In this study, we performed a comparative phosphoproteomic analysis to investigate the molecular mechanisms of SA-induced defense response in different rice cultivars. A total of 29 SA-responsive phosphoproteins were successfully identified by MAIDL-TOF/TOF analysis. Phosphoproteins involved in photosynthesis, antioxidative enzymes, molecular chaperones showed similar changes in the two cultivars, while phosphoproteins related to protein synthesis and degradation, defense, amino acid metabolism, and carbohydrate metabolism were differentially expressed. Furthermore, phosphorylation within four identified phosphoproteins was validated by NanoLC-MS/MS analysis, and phosphorylated regulation of three important enzymes (CCR, PGAM and APX) was verified by activity determination. To best of our knowledge, it is the first report to measure rice phosphoproteomic changes induced by SA, which may broaden our understanding of SA-responsive mechanisms in rice.

## Methods

### Chemicals

The following chemical reagents were used in this study: SA (Sigma-Aldrich, St. Louis, MO, USA); Immobiline™ DryStrip pH 4–7 NL, 18 cm and IPG buffer pH 4–7 (GE Healthcare, Uppsala, Sweden); Pro-Q Diamond phosphoprotein gel stain (Molecular Probes, Eugene, OR, USA); Glycine, 1.5 mol/L Tris-HCl buffer pH 8.8, 30% acrylamide/bis solution (37.5:1), and overlay agrose (Bio-Rad, Hercules, CA, USA).

### Plant materials

Two rice near isogenic lines (*Oryza sativa indica*) were obtained from the International Rice Research Institute, including C101LAC carrying the resistance gene *Pi-1* against *M. oryzae*, and background line CO39 carrying no known resistance gene. Rice seedlings were sprayed with 0.1 mM SA solution (containing 0.02% v/v Tween 20) at the four-leaf stage [7]. The fourth leaves were harvested at 12 and 24 h after SA treatment. Spraying with sterilized water containing 0.02% v/v Tween 20 served as blank control. The leaves were sampled by freezing in liquid nitrogen, and stored at − 80 °C before assessment.

### Phosphoproteome enrichment, 2DE and gel analysis

Total proteins were extracted essentially from 5 g of rice leaf samples by using a PEG-mediated prefractionation method [45]. Enrichment of phosphorylated proteins follows the well-established Al (OH)_3_-MOAC method [30]. The protein content was determined using the coomassie blue dye-binding method, with BSA as the standard [46]. Three replicates with different pools of leaf samples were performed, and all the procedures were carried out at 4 °C.

2DE follows the established protocol [47]. Phosphoproteins were visualized with Pro-Q Diamond fluorescent gel stain according to the methods described previously [48], before imaging with a Typhoon Trio Variable Mode Imager (GE Healthcare, Uppsala Sweden). PDQuest software (Version 8.0, Bio-Rad, Hercules, CA, USA) was used for quantitative analysis with gel spots, including spot detection, measurement, matching and calculation. Each phosphoprotein sample was analyzed by 2DE for at least three times. The protein spots showing ≥1.5-fold increase or decrease in all three biological repeats were selected as putative differentially regulated phosphoproteins.

### Phosphoprotein identification by MALDI-TOF/TOF MS

The differentially expressed phosphoprotein spots were manually excised from the gels, before in-gel digestion [7]. The peptides were subsequently analyzed using the ABI 4800 Proteomics Analyzer MALDI-TOF/TOF (Applied Biosystems, Foster City, CA). Database search was performed in the *Oryza sativa* database (Uniprot, v.2016.08.24) using the MASCOT search engine 2.2 (Matrix Science, Ltd.) with GPS-Explorer Software 3.6 (Applied Biosystems). The parameter settings were as following: peptide mass tolerance: 100 ppm; fragment tolerance: ±0.3 Da; protein score C.I.%: ≥95%; total ion score C.I.%: ≥95% and significance threshold: *p* < 0.05. Besides, to eliminate the redundancy of proteins that appeared in the database under different names and accession numbers, the single-protein member belonging to the species of *O. sativa* or others with the highest protein score (top rank) was singled out from the multi-protein family.

### Identification of phosphorylation sites by NanoLC-MS/MS

Total proteins extraction from rice leaves was performed using a PEG-mediated prefractionation method [30], and protein was digested with trypsin following the FASP method [49]. The trypsin-digested peptide mixture was then loaded onto aliquot of titanium dioxide (TiO_2_) beads (5 μm Titansphere, GL Sciences, Japan), which were then collected by centrifugation after washing twice with 30 mg/mL DHB (2,5-dihydroxybenzoic acid) buffer. The beads were further washed for twice with 60% ACN/0.1% TFA and 0.1% TFA respectively, before elution with a 60% ACN/4% ammonium solution. NanoLC-MS/MS was performed with a Q Exactive MS (Thermo Finnigan) equipped with Easy nLC1000 (ThermoFisher, San Jose, CA). The peptide mixture was seperated on a C18-reversed phase column with a flow rate of 250 nL/min over 240 min. Peptides were analyzed by MS/MS in positive ion mode, and the MS/MS spectra search was performed against the Uniprot_*Oryza* database (v.2018.02.27), using Mascot 2.2 engine. Proteome Discoverer 1.3 (Thermo Electron, San Jose, CA) was used for identification of phosphorylation peptides, with the threshold setting as pRS score above 50 indicating a good PSM (Peptide Spectrum Matches) and pRS probabilities above 75% indicating a truly phosphorylated site.

### Determination of enzyme activities and reactive oxygen species

The fourth leaves were sampled at 12 and 24 h after SA treatment. Enzymatic activity of APX (ascorbate peroxidase), CCR (cinnamoyl-CoA reductase) activity, and phosphoglycerate mutase (PGAM) was assayed respectively following the established protocols [50–52]. Hydrogen peroxide (H_2_O_2_) follows Brennan and Frenkel’s method [53]. The rate of superoxide (O_2_^.−^) production was measured based on nitroblue tetrazolium (NBT) reduction [54]. Determination of malondialdehyde (MDA) content follows the previously described method [55], using the following formula to calculate MDA content (*C*): *C* (μmol/L) = 6.45 (*A*_532_- *A*_600_)-0.56 *A*_450_.

### Quantitative real-time PCR (qRT-PCR) analysis

Total RNA isolation from rice leaves was performed using Eastep^@^ Super Total RNA Extraction Kit (Promega, Shanghai, China). The first-strand cDNA was prepared from 2 μg of normalized total RNA, with FastKing RT Kit (with gDNase) (Tiangen Biotech, Beijing, China). Gene-specific primers used for selected gene transcription assessment were designed using the Primer 5.0 software and were listed in (Additional file 4: Table S4). The tubulin gene (Uniprot Accession No. Q58G87) was used as reference. qRT-PCR was conducted on a CFX Coxnnect™ Real-Time System (Bio-Rad, Hercules, CA, USA) with the iTaq™ SYBR® Green Supermix (Bio-Rad, Hercules, CA, USA) according to the manufacturer’s protocol. Three independent biological replicates were performed for each gene. Relative transcript levels for each gene were calculated by 2^−△△Ct^ method [56].

### Statistical analysis

Means ± standard error (SE) was derived from three biological replicates. The data were analyzed using the one-way analyses of variance (ANOVA) and the significant differences were determined as *p* ≤ 0.05, by the Duncan’s test using SPSS software (version 19.0, SPSS Inc., Chicago, IL, USA).

## Supplementary information

**Additional file 1: Figure S1.** Representative 2DE patterns of phosphoproteins from rice leaves treated with MQ water (as the control) and SA. **Figure S2** All additional 2DE gels of rice phosphoproteins were shown as replicate gels. **Figure S3.** Close-up views of the regions of 2DE gels showing all SA-responsive phosphoprotein spots in two rice cultivars. **Figure S4.** Quantitative analysis of the SA-responsive phosphoproteins in rice leaves. **Figure S5.** Identification of spot 11 by MALDI-TOF/TOF MS. **Figure S6.** The functional category distribution of the 29 SA-responsive phosphoproteins. **Figure S7.** The MS/MS spectra of representative phosphorylated peptides of ELLS*YEYDGDEVPIVAGSALK, corresponding to Elongation factor Tu (Q6ZI53).

**Additional file 2: Table S1.** Phosphopeptides and their phosphorylation sites (marked by lowercase letters of the amino acid residue) identified by NanoLC-MS/MS analysis.

**Additional file 3: Table S2.** Rice phosphoproteins identified by NanoLC-MS/MS analysis.

**Additional file 4: Table S3.** Mapping of SA-responsive phosphoproteins with the NanoLC-MS/MS data. **Table S4.** Gene-specific primers designed for qRT-PCR.

## Data Availability

The data generated or analyzed during this study are included in this published article and its supplementary information files.

## Abbreviations

2DE: Two-dimensional gel electrophoresis
APX: Ascorbate peroxidase
CCR: Cinnamoyl-CoA reductase
IEF: Isoelectric focusing
IPG: Immobiline pH gradien
LC: Liquid chromatography
MOAC: Metal oxide affinity chromatography
MS: Mass spectrometer
MS/MS: Tandem mass spectrometry
PEG: Polyethylene glycol
PGAM: Phosphoglycerate mutase
Pro-Q DPS: Pro-Q diamond phosphoprotein stain
PTMs: Post-translational modifications
qRT-PCR: Quantitative real-time PCR
ROS: Reactive oxygen species
SA: Salicylic acid
