# Supplementary material for: Comparative phosphoproteomic analysis of blast resistant and susceptible rice cultivars in response to salicylic acid
Source: BMC Plant Biol. 2019 Oct 28;19:454. doi: 10.1186/s12870-019-2075-5 (PMC6819546; doi:10.1186/s12870-019-2075-5)
Supplement: Supplementary file 1 — Additional file 1: Figure S1. Representative 2DE patterns of phosphoproteins from rice leaves treated with MQ water (as the control) and SA. Figure S2 All additional 2DE gels of rice phosphoproteins were shown as replicate gels. Figure S3. Close-up views of the regions of 2DE gels showing all SA-responsive phosphoprotein spots in two rice cultivars. Figure S4. Quantitative analysis of the SA-responsive phosphoproteins in rice leaves. Figure S5. Identification of spot 11 by MALDI-TOF/TOF MS. Figure S6. The functional category distribution of the 29 SA-responsive phosphoproteins. Figure S7. The MS/MS spectra of representative phosphorylated peptides of ELLS*YEYDGDEVPIVAGSALK, corresponding to Elongation factor Tu (Q6ZI53). [file 12870_2019_2075_MOESM1_ESM.docx]

**Fig. S1** Representative 2DE patterns of phosphoproteins from rice leaves treated with MQ water (as the control) and SA. **a**, CO39-control-12 h; **b,** CO39-SA-12 h; **c**, C101LAC-control-12 h; **d**, C101LAC-SA-12 h; **e**, CO39-control-24 h; **f,** CO39-SA-24 h; **g**, C101LAC-control-24 h; **h**, C101LAC-SA-24 h. Twelve and twenty-four hours after SA treatment, total phosphoproteins were prepared and an aliquot (150 μg) were loaded on an 18-cm IPG strip with a linear gradient of pH 4-7 for IEF, following electrophoresis of 12% SDS-PAGE. Phosphoprotein spots were visualized by staining with Pro-Q Diamond. Arrows indicate the positions of differentially expressed phopshoprotein spots compared with their respective controls. Numbered spots correspond to phophoproteins listed in Table 1.

**Fig. S2** All additional 2DE gels of rice phosphoproteins were shown as replicate gels. **a2 and a3**, CO39-control-12 h; **b2 and b3,** CO39-SA-12 h; **c2 and c3**, C101LAC-control-12 h; **d2 and d3**, C101LAC-SA-12 h; **e2 and e3**, CO39-control-24 h; **f2 and f3,** CO39-SA-24 h; **g2 and g3**, C101LAC-control-24 h; **h2 and h3**, C101LAC-SA-24 h.

**Fig. S3** Close-up views of the regions of 2DE gels showing all SA-responsive phosphoprotein spots in two rice cultivars. Arrows indicate phosphoproteins whose expression changed in response to SA treatment. The relative locations of these phosphoprotein spots are indicated in Fig. S1. Symbol: CK, rice control treated with water; SA, rice treated with SA.

**Fig. S4** Quantitative analysis of the SA-responsive phosphoproteins in rice leaves. The protein intensity of the phosphoprotein spots were calculated with PDQuest 8.0 software. **a** 12 h post SA treatment , and **b** 24 h post SA treatment . Values were the means (±SE) of protein intensity on gels from three independent experiments. The letters above the bars of the same phosphoprotein spot indicate a statistically significant difference in protein expression at 0.05 level. Symbol: CK, rice control treated with water; SA, rice treated with SA.

**Fig. S5** Identification of spot 11 by MALDI-TOF/TOF MS. The protein excised from 2DE gels was digested with trypsin, and the resulting peptides were analyzed using the 4800 Proteomic Analyzer. **a**, The MS spectra. The matched peptides and their corresponding peaks are listed in the map. The ion 1084.58 marked with an asterisk was analyzed by MS/MS. **b**, MS/MS spectra of ion 1084.58. After database searching, the protein was identified as Probable glutamyl endopeptidase, chloroplastic.

**Fig. S6** The functional category distribution of the 29 SA-responsive phosphoproteins.

**Fig. S7** The MS/MS spectra of representative phosphorylated peptides of ELLS*YEYDGDEVPIVAGSALK, corresponding to Elongation factor Tu (Q6ZI53). The asterisk indicates phosphorylation on the right side of the serine residue.
