# Supplementary material for: Comparative phosphoproteomic analysis of blast resistant and susceptible rice cultivars in response to salicylic acid
Source: BMC Plant Biol. 2019 Oct 28;19:454. doi: 10.1186/s12870-019-2075-5 (PMC6819546; doi:10.1186/s12870-019-2075-5)
Supplement: Supplementary file 4 — Additional file 4: Table S3. Mapping of SA-responsive phosphoproteins with the NanoLC-MS/MS data. Table S4. Gene-specific primers designed for qRT-PCR. [file 12870_2019_2075_MOESM4_ESM.docx]

**Table S3** Mapping of SA-responsive phosphoproteins with the nanoLC-MS/MS data

| Spot No.^a^ | Matched proteins^b^ | Peptide sequence and  phsophorylation sites^c^ | pRS score | Charge | Mr (Da) | Phosphorylated residues |
| --- | --- | --- | --- | --- | --- | --- |
| 24, 25 | Q9ATK9 | FADDEDLQ*EWR | 105 | 2 | 1590.619 | Serine |
| 30 | Q6ZI53 | ELLS*YEYDGDEVPIVAGSALK | 67 | 2 | 2435.139 | Serine |
| 31, 32 | Q6Z8F4 | HADFPG*SNNGTGLFQTIVGLK | 120 | 2 | 44865 | Serine |
| 34 | B0FFP0 | TD*EGGFESDAVATANILESSAPVVGGK | 104 | 2 | 2788.27 | Serine |

^a^ Spot numbers are according to 2DE gels as shown in Fig. 1.

^b^ Uniprot Accession No.

^c^ The asterisk indicates phosphorylation sites on the right side of the serine residue.

**Table S4** Gene-specific primers designed for qRT-PCR

| Protein Spot no. ^a^ | Protein name | Gene name | Uniprot  Accession No. | Forward primer (5’→3’) | Reverse primer (5’→3’) | Length of Product Size (bp) |
| --- | --- | --- | --- | --- | --- | --- |
| 17 | Glyceraldehyde-3-phosphate dehydrogenase | OsI_09835 | A2XC18 | CAAGCTGATCAAGGTCGTCTC | GGAGCAGTGATGATGACCTTC | 159 |
| 23 | Putative chaperonin 60 beta | Os06g0114000 | Q9LWT6 | TTCAGGTGGGAGCACAAAC | GCTCATCATTCTCCAGGTTGT | 177 |
| 28 | Eukaryotic initiation factor 4A-1 | Os06g0701100 | P35683 | CCCGTCAAAGATCCAAGTT | GAGTGTGACAAGCTTCCATTC | 181 |
| 30 | Elongation factor Tu | OJ1126_D09.31-1 | Q6ZI53 | GGTTACCGCCCTCAGTTCTA | CCTCATTCCCTGCTCACAA | 162 |
| 31 | Phosphoribulokinase | Os02g0698000 | Q6Z8F4 | GACTGGCAGGAAGGAGAAAG | GGGATGTAAGCCCTCAATG | 196 |
| 33 | L-ascorbate peroxidase 1, cytosolic | APX1 | B7E6Z4 | CTGATGCTACCAAGGGTTCTG | TGTCCAAGGTCCCTCAAAAC | 146 |
| Reference gene | Polyubiquitin 3 | UBQ3 | Q58G87 | TGAGTTCTCCGATACGATTGAC | TGTTATAATCCGCGAGAGTACG | 130 |

^a^ Spot numbers are according to 2DE gels as shown in Fig. 1.
